# A case control study of subjective sleep characteristics and polycystic ovary syndrome

**DOI:** 10.1038/s41598-026-50477-3

**Published:** 2026-04-29

**Authors:** Xiulian Zhuang, Shipeng Zhang, Rui Fu, Chang Cai, Jiali Yin, Wenhui Shi, Jiaqu Cen, Xiaoli Ji

**Affiliations:** 1 Clinical Medical College, Chengdu University of Traditional Chinese Medicine, Chengdu, Sichuan China; 2 Fushun County Hospital of Traditional Chinese Medicine, Zigong, Sichuan China; 3 Hospital of Chengdu University of Traditional Chinese Medicine, Chengdu, Sichuan China

**Keywords:** Polycystic ovary syndrome, Insulin resistance, Sleep quality, Metabolic syndrome, Case-control study, Diseases, Endocrinology, Health care, Medical research, Risk factors

## Abstract

To investigate the subjective sleep characteristics of patients with polycystic ovary syndrome (PCOS) and their association with the syndrome. A case-control study was conducted, enrolling 136 patients with PCOS and 164 non-PCOS controls recruited from the gynecology outpatient department between February and October 2025. Sleep quality was assessed using a simplified questionnaire derived from core items of the Pittsburgh Sleep Quality Index. Multivariate logistic regression analysis was performed to evaluate the association between sleep problems and PCOS, with adjustment for age, body mass index (BMI), and other potential confounders. The PCOS group exhibited significantly higher prevalences of difficulty falling asleep (51.5% vs. 32.3%), non-restorative sleep (51.5% vs. 32.9%), and self-reported poor sleep quality (66.2% vs. 53.7%) compared with the control group (all *p* < 0.05). The prevalence of frequent dreaming was significantly higher in the PCOS group than in the control group (39.0% vs. 28.0%, *p* = 0.045, chi-square test). However, in the correlation analysis, frequent dreaming was not significantly associated with PCOS status (*p* > 0.05). After adjustment for potential confounders, participants with moderate and high sleep problem scores demonstrated a significantly increased risk of PCOS (moderate group: adjusted odds ratio [aOR] = 1.845, 95% CI: 1.025–3.320; high group: aOR = 2.286, 95% CI: 1.054–4.959). Subjective sleep disturbances are highly prevalent among patients with PCOS, particularly characterized by difficulty falling asleep, non-restorative sleep, and perceived poor sleep quality. The severity of these sleep disturbances is independently associated with an increased risk of PCOS. Subjective sleep assessment should be incorporated into the clinical management of PCOS.

**Supplementary Information:**

The online version contains supplementary material available at 10.1038/s41598-026-50477-3.

## Introduction

 Polycystic ovary syndrome (PCOS) is a common endocrine and metabolic disorder affecting women of reproductive age worldwide, with an estimated prevalence of approximately 5–20%^1–3^. This condition is diagnosed based on the presence of hyperandrogenism, ovulatory dysfunction, and/or polycystic ovarian morphology. Clinical manifestations are heterogeneous and include hirsutism, acne, menstrual irregularities, infertility, an elevated body mass index (BMI), insulin resistance (IR), and dyslipidemia^4–8^. Patients with PCOS also face an increased risk of miscarriage and pregnancy-related complications during gestation (e.g., gestational hypertension/preeclampsia, gestational diabetes, and preterm birth)^9–12^. Moreover, psychological comorbidities, particularly depression and anxiety, are highly prevalent in women with PCOS^13,14^. PCOS imposes substantial burdens on the reproductive, metabolic, and mental health of women worldwide.

The etiology of PCOS remains incompletely understood, with contributing factors including genetic predisposition, environmental influences, and unhealthy lifestyle behaviors^15^. Recent studies have highlighted the important role of gut microbiota dysbiosis in the pathogenesis and progression of PCOS^16^. The convergence of multiple etiological factors induces a pathological state characterized by hormonal imbalance, IR, and chronic low-grade inflammation, thereby driving the pathological process of PCOS. This cascade of disturbances constitutes a key pathological basis for impaired reproductive health and is considered to potentially compromise multiple critical reproductive processes, including oocyte quality, embryonic developmental potential, and endometrial receptivity, ultimately leading to reduced overall reproductive potential in women with PCOS^17–20^.

At present, there is no definitive cure for this condition, and clinical management primarily focuses on lifestyle interventions, particularly weight management, with pharmacological therapies as needed to regulate menstrual cycles and improve metabolic function, and with ovulation induction or assisted reproductive technologies employed to manage infertility when indicated^21,22^. However, existing therapeutic approaches have notable limitations: some non-obese individuals still develop PCOS, long-term hormonal therapy is associated with potential risks, and ovulation induction is not effective for all patients with infertility^23–25^. Notably, beyond metabolic issues, sleep problems are highly prevalent among patients with PCOS. Moreover, existing research on the relationship between sleep and PCOS has predominantly focused on obesity-related obstructive sleep apnoea (OSA), making it difficult to disentangle the association from obesity as a confounder^26^. Clinical observations and existing research indicate that even non-overweight patients with PCOS frequently report difficulty initiating sleep and poor sleep quality^27^. Although it has been suggested that sleep deprivation and circadian rhythm disruption may contribute to the progression of PCOS by exacerbating endocrine dysfunction, IR, and low-grade inflammation^28^. However, systematic investigations examining the associations between specific sleep dimensions—such as quality, duration, and circadian rhythm—and PCOS remain relatively scarce.

Based on the foregoing considerations, this study aims to conduct a preliminary investigation of sleep patterns among patients with PCOS. Using established instruments such as the Pittsburgh Sleep Quality Index (PSQI), together with a simplified questionnaire tailored to the study objectives, we systematically assessed dimensions including sleep duration, circadian rhythm, and subjective sleep quality. This approach aims to characterize sleep features in the PCOS population and provide a preliminary analysis of their associations with PCOS status.

## Methods

### Study design, population and sample size

This study recruited patients attending the gynaecology outpatient department of the Affiliated Hospital of Chengdu University of Traditional Chinese Medicine between February and October 2025 as survey participants. The required sample size was calculated using the following formula^29^.

1 $$Sample\:size=\frac{r+1}{r}\frac{(Z\beta+Z\alpha/2)^2\:P(1-P)}{(P1-P2)^2}$$

In this study, r denotes the case-control ratio, set at 1 to indicate an equal number of cases and controls. Zα/2 represents the standard normal variable at a significance level of 0:05/2, equating to 1.96, while Zβ denotes the value corresponding to 90% power, which is 1.28. P1 denotes the case proportion, i.e., the previously reported prevalence of PCOS, which stands at 5.61%^30^. P2 denotes the proportion in the control group. Following consultation with statisticians, the proportion of reproductive-aged gynaecological patients without PCOS was assumed to be 0.27. Sample size calculations indicated that 126 cases were required. To ensure adequate representativeness and statistical validity of both the case and control groups, a total of 300 participants was planned for enrolment. All completed questionnaires were reviewed for completeness, and any questionnaires with missing responses were excluded from the analysis. Ultimately, 136 valid questionnaires were obtained from the case group and 164 from the control group.

**Fig. 1:**
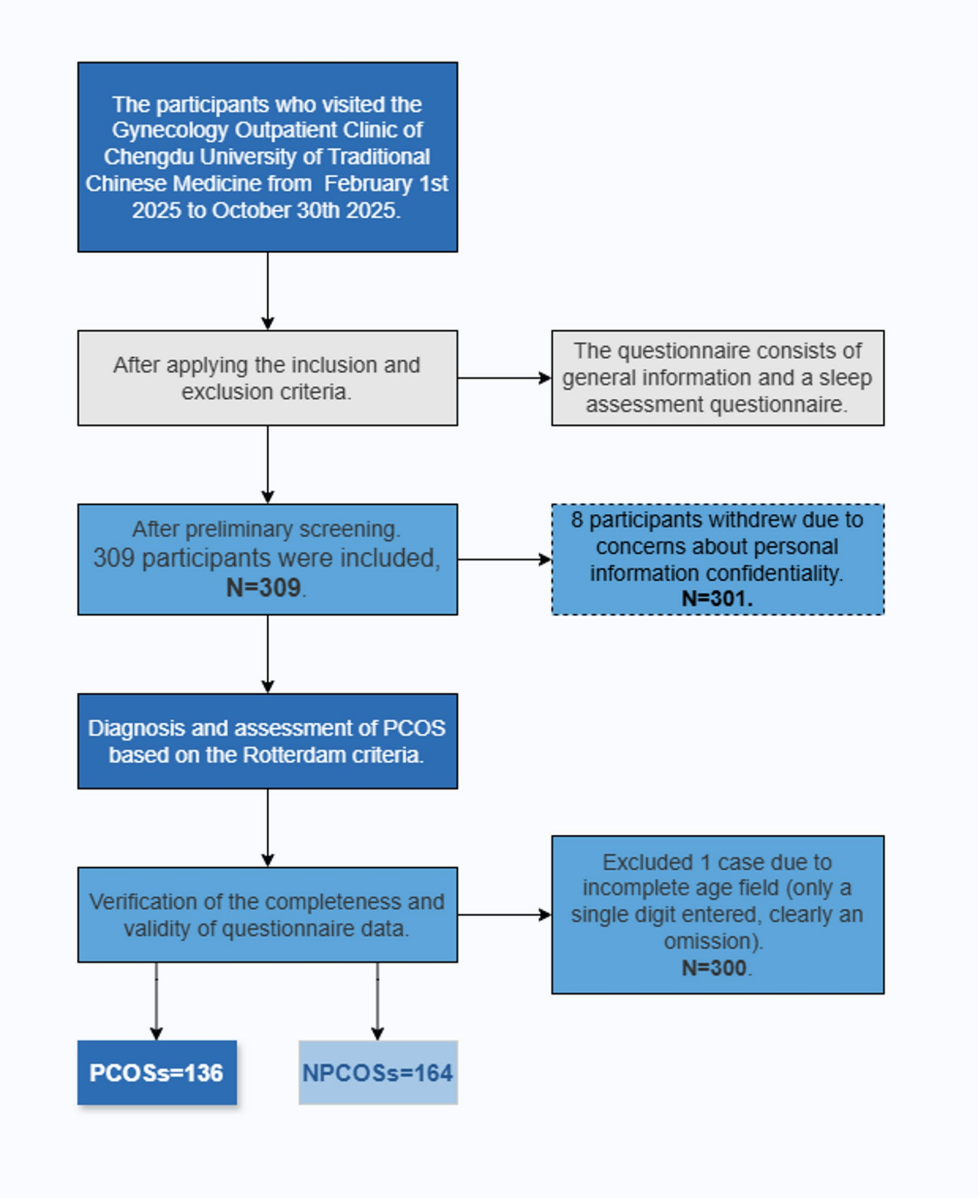
Flowchart of participant recruitment and selection.

### Inclusion and exclusion criteria

Women aged 18–49 years attending gynaecological outpatient clinics were eligible for inclusion in this study. Case group (PCOS group): Participants met the 2003 Rotterdam diagnostic criteria and were confirmed by a gynaecologist to have PCOS, requiring fulfilment of at least two of the following three criteria: (i) infrequent ovulation or anovulation; (ii) clinical and/or biochemical evidence of hyperandrogenism; and (iii) ultrasonographic evidence of polycystic ovarian morphology. Other relevant conditions, including congenital adrenal hyperplasia, Cushing’s syndrome, and ovarian or adrenal tumours, were excluded. Participants were required to be able to understand and complete the study questionnaire and to provide written informed consent with voluntary participation.

Control group: (1) Patients attending the same gynaecological outpatient clinic during the same period, presenting with complaints of menstrual irregularities, lower abdominal discomfort, or routine check-ups, but clinically assessed and definitively excluded from a diagnosis of PCOS; (2) Confirmed non-PCOS following systematic clinical evaluation; (3) Able to comprehend and complete the study questionnaire; (4) Provided informed consent and voluntarily participated in the study.

The exclusion criteria were as follows: (1) inability to accurately recall sleep patterns during the preceding month; (2) use of hormonal medications (e.g., oral contraceptives, glucocorticoids) or medications known to affect sleep, metabolic, or endocrine function (e.g., antidepressants, antipsychotics, sedative-hypnotics) within the preceding three months; (3) presence of severe endocrine disorders (e.g., hyperthyroidism, hypothyroidism, Cushing’s syndrome, type 1 diabetes), autoimmune diseases, severe hepatic or renal insufficiency, or malignant tumours; (4) history of ovarian surgery (e.g., ovarian wedge resection or drilling) or hysterectomy; (5) diagnosed psychiatric or psychological disorders (e.g., depression, anxiety disorders, bipolar disorder) or cognitive impairment that precluded completion of the questionnaire; and (6) pregnancy, breastfeeding, or plans for pregnancy within the preceding three months.The questionnaire did not directly collect medical information for exclusion criteria items (2)–(6). Item (1) was self-assessed by participants prior to questionnaire completion, whereas the remaining items were derived from clinical evaluation and medical record review, as determined by gynecologists during outpatient consultations.

The study protocol was approved by the Ethics Committee of the Affiliated Hospital of Chengdu University of Traditional Chinese Medicine (2024KL-051-01). All participants provided written informed consent after being informed of the study objectives and procedures. Of note, the PCOS status variable in all analyses was based on gynecologists’ clinical evaluations (see inclusion criteria), while the self-report item “Have you been clinically diagnosed with polycystic ovary syndrome (PCOS)?” was employed solely for cross-checking and quality control purposes.

### Questionnaire design

#### General questionnaire

This section was designed to collect baseline demographic and clinical information, including age, ethnicity, occupation, educational attainment, place of residence (with consideration of environmental pollution levels), height and weight (used to calculate BMI) and individual diagnostic status. Smoking and alcohol consumption habits were also assessed and recorded as potential confounding factors.

#### Assessment of sleep status

This module constituted the core of the questionnaire and was designed to systematically assess participants’ subjective sleep quality and sleep behaviours. It comprised eight items, based on the core dimensions of the PSQI, with simplifications reflecting common clinical complaints. Specific assessment components included:

1. **Sleep rhythm**: average daily bedtime, rated on a five-point scale from ‘Before 10:00 pm’ to ‘After 1:00 am’ (0–4 points);
2. **Sleep duration**: average daily sleep duration, rated from ‘>8 h’ to ‘<6 h’ (0–3 points), with shorter durations corresponding to higher scores;
3. **Sleep quality and disturbances**: included difficulty falling asleep, nocturnal awakenings, early morning awakenings with inability to return to sleep, non-restorative sleep, and vivid dreaming. Each item was scored dichotomously (‘Yes’ = 1, ‘No’ = 0);
4. **Subjective sleep assessment**: assessed by the item “Do you consider your recent sleep quality to be good?” with dichotomous responses (‘Yes’ = 1, ‘No’ = 0).

The sum of all item scores constituted the total sleep problem score (range: 0–13), with higher scores indicating more severe sleep problems. Based on quartile ranges, scores of 1–4 defined the low-score group, 5–9 the medium-score group, and 10–12 the high-score group. The questionnaire demonstrated good reliability (Kaiser–Meyer–Olkin [KMO] = 0.811) and internal consistency (Cronbach’s α = 0.752), with satisfactory construct validity.

### Statistical methods

Data analysis was performed using SPSS version 31.0 software (IBM, Armonk, New York, United States). The reliability and validity of the scale were assessed using Cronbach’s α, Bartlett’s test of sphericity, and the KMO test. Continuous variables are presented as mean ± standard deviation, and differences between the case and control groups were compared using independent samples t-tests. Categorical variables are presented as frequency (n) and percentage (%), and group comparisons were performed using the chi-square test.

To explore the association between sleep-related items and PCOS status, Spearman’s rank correlation analysis was first conducted between individual sleep scores and PCOS status. To further quantify the relationship between the overall burden of sleep problems and the risk of PCOS, the total sleep problem score was used as the core independent variable in a binary logistic regression model to examine its association with PCOS status. The regression analysis calculated odds ratios (ORs) with 95% confidence intervals (95% CIs), adjusting for potential confounders such as age, occupation, educational level, BMI, smoking history, and drinking history. All statistical tests were two-sided, and a p-value < 0.05 was considered statistically significant.

## Results

### General findings

A total of 309 reproductive-aged women attending outpatient clinics were assessed in this study. During the study, eight participants withdrew due to concerns regarding personal information confidentiality, and one participant was excluded due to an incomplete age field (only a single digit was entered, clearly an omission). Ultimately, 300 participants were included in the analysis, comprising 136 in the PCOS group and 164 in the non-PCOS group. The sample screening process is illustrated in Fig. 1.

Baseline characteristics of the two participant groups are summarized in Table 1. No statistically significant differences were observed between the PCOS and non-PCOS groups in terms of age, height, ethnicity, or environmental pollution exposure (*p* > 0.05). Weight and BMI were significantly higher in the PCOS group than in the non-PCOS group (*p* < 0.001). Significant differences were also observed between the groups in occupational distribution (*p* < 0.001) and educational attainment (*p* = 0.001). No significant differences were found in smoking (*p* = 0.114) or alcohol consumption (*p* = 0.775).

Regarding sleep patterns, no statistically significant differences were observed between the groups in sleep onset latency (*p* = 0.442), total sleep duration (*p* = 0.077), nocturnal awakenings (*p* = 0.336), or early morning awakening with inability to return to sleep (*p* = 0.465). In contrast, significant differences were observed in difficulty falling asleep (*p* < 0.001), persistent fatigue upon waking (*p* = 0.001), vivid dreaming (*p* = 0.045), and perceived poor sleep quality (*p* = 0.028). When item responses were scored and summed, the PCOS group exhibited a significantly higher cumulative sleep disturbance score (*p* = 0.004).

**Table 1:** Baseline characteristics of study participants by group.

		PCOSs	NPCOSs	Statistic (t/χ²)	*p*
Age (M ± SD)		26.65 ± 5.40	27.38 ± 6.23	−1.072	0.285
Height (M ± SD)		1.60 ± 0.05	1.61 ± 0.06	−0.842	0.400
Weight (M ± SD)		61.77 ± 12.65	55.35 ± 9.59	4.993	**< 0.001** ^***^
Body mass index (M ± SD)		24.02 ± 4.61	21.39 ± 3.41	5.686	**< 0.001** ^***^
Ethnic group (N, %)	Han ethnicity	133 (97.8%)	153 (93.3%)	3.386	0.066
	Ethnic minorities	3 (2.2%)	11 (6.7%)		
Occupation^a^ (N, %)	Professional/technical	40 (29.4%)	73 (44.5%)	28.389	**< 0.001** ^***^
	Service sector	21 (15.4%)	11 (6.7%)		
	Student	30 (22.1%)	57 (34.8%)		
	Homemaker/freelancer	11 (8.1%)	7 (4.3%)		
	Manager/civil servant	13 (9.6%)	10 (6.1%)		
	Other	21 (15.4%)	6 (3.7%)		
Degree of education^b^ (N, %)	Junior high school and below	7 (5.1%)	4 (2.4%)	13.60	**0.001** ^**^
	Senior high school	17 (12.5%)	4 (2.4%)		
	University and above	112 (82.4%)	156 (95.1%)		
Enviromental pollution (N, %)	Urban	110 (80.9%)	128 (78.0%)	0.651	0.722
	Town	19 (14.0%)	24 (14.6%)		
	Rural	7 (5.1%)	12 (7.3%)		
Smoking (N, %)	Yes	123 (90.4%)	156 (95.1%)	2.502	0.114
	No	13 (9.6%)	8 (4.9%)		
Drinking (N, %)	Yes	81 (59.6%)	95 (57.9%)	0.082	0.775
	No	55 (40.4%)	69 (42.1%)		
Usual Bedtime^c^ (N, %)	Before 10:00 PM	1 (0.7%)	1 (0.6%)	0.592	0.442
	10:00–11:00 PM	25 (18.4%)	23 (14.0%)		
	11:00 PM-12:00 AM	48 (35.3%)	73 (44.5%)		
	12:00–1:00 AM	45 (33.1%)	59 (36.0%)		
	After 1:00 AM	17 (12.5%)	8 (4.9%)		
Sleep Duration^c^ (N, %)	< 6 h	4 (2.9%)	4 (2.4%)	3.131	0.077
	6–7 h	37 (27.2%)	63 (38.4%)		
	7–8 h	63 (46.3%)	68 (41.5%)		
	> 8 h	32 (23.5%)	29 (17.7%)		
Difficulty Falling Asleep (within 30 min) (N, %)	Yes	70 (51.5%)	53 (32.3%)	11.275	**< 0.001** ^***^
	No	66 (48.5%)	111 (67.7%)		
Nocturnal Awakenings (N, %)	Yes	39 (28.7%)	39 (23.8%)	0.926	0.336
	No	97 (71.3%)	125 (76.2%)		
Early Morning Awakening (with inability to resume sleep) (N, %)	Yes	46 (33.8%)	49 (29.9%)	0.535	0.465
	No	90 (66.2%)	115 (70.1%)		
Non-restorative Sleep (N, %)	Yes	70 (51.5%)	54 (32.9%)	10.543	**0.001** ^**^
	No	66 (48.5%)	110 (67.1%)		
Frequent dreams (N, %)	Yes	53 (39.0%)	46 (28.0%)	4.011	**0.045** ^*^
	No	83 (61.0%)	118 (72.0%)		
Subjective Sleep Quality (over the past month) (N, %)	Yes (Good)	46 (33.8%)	76 (46.3%)	4.828	**0.028** ^*^
	No (Poor)	90 (66.2%)	88 (53.7%)		
Sleep Problem Score (M ± SD)		6.98 ± 2.82	6.05 ± 2.73	2.869	**0.004** ^**^

Notes: Continuous variables are presented as Mean ± SD and compared using independent samples t-test; categorical variables are presented as n (%) and compared using the Chi-square test, unless otherwise specified. *p < 0.05, **p < 0.01, ***p < 0.001.

BMI: body mass index.

a. For ‘Occupation’, the categories ‘Manual Laborer’, ‘Unemployed’, and ‘Other’ were merged into ‘Other Occupations’.

b. For ‘Education Level’, the Pearson chi-square test is reported (one cell [16.7%] had an expected count of 4.99).

c. For the ordered categorical variables ‘Usual Bedtime’ and ‘Sleep Duration’, the linear-by-linear association test was used.

### Relationship between sleep patterns and PCOS

Spearman’s rank correlation analysis was employed to examine the association between sleep patterns and PCOS status (Table 2). The analysis revealed significant positive correlations between PCOS and difficulty falling asleep (*p* = 0.001) as well as fatigue upon waking (*p* = 0.001). Participants reporting poor subjective sleep quality exhibited a higher likelihood of PCOS (*p* = 0.028). No statistically significant correlations were observed between PCOS status and sleep onset time, sleep duration, nocturnal awakenings, early morning awakening, or vivid dreaming (*p* > 0.05).

**Table 2:** Spearman’s rank correlation analysis between polycystic ovary syndrome and sleep-related variables.

Items		Polycystic ovarian syndrome	Usual bedtime	Sleep duration	Difficulty falling asleep	Nocturnal awakenings	Early morning awakening	Non-restorative sleep	Frequent dreams	Subjective sleep quality
Polycystic ovarian syndrome	Spearman correlation	1								
	p value	/								
Usual bedtime	Spearman correlation	0.039	1							
	p value	0.499	/							
Sleep duration	Spearman correlation	0.109	0.636^***^	1						
	p value	0.059	< 0.001	/						
Difficulty falling asleep	Spearman correlation	0.187^**^	0.522^***^	0.384^***^	1					
	p value	0.001	< 0.001	< 0.001	/					
Nocturnal awakenings	Spearman correlation	0.056	0.378^***^	0.407^***^	0.221^***^	1				
	p value	0.337	< 0.001	< 0.001	< 0.001	/				
Early morning awakening	Spearman correlation	0.042	0.634^***^	0.442^***^	0.370^***^	0.266^***^	1			
	p value	0.466	< 0.001	< 0.001	< 0.001	< 0.001	/			
Non-restorative sleep	Spearman correlation	0.187^**^	0.164^**^	0.139^*^	0.146^*^	0.166^**^	0.156^**^	1		
	p value	0.001	0.004	0.016	0.012	0.004	0.007	/		
Frequent dreams	Spearman correlation	0.108	0.047	0.060	0.060	0.008	0.061	0.166^**^	1	
	p value	0.061	0.414	0.304	0.300	0.884	0.295	0.004	/	
Subjective sleep quality	Spearman correlation	0.127^*^	0.451^***^	0.288^***^	0.409^***^	0.197^***^	0.359^***^	0.254^***^	0.186^**^	1
	p value	0.028	< 0.001	< 0.001	< 0.001	< 0.001	< 0.001	< 0.001	0.001	/

*p < 0.05, **p < 0.01, ***p < 0.001.

To investigate the independent association between sleep problem severity and PCOS risk, a multivariate logistic regression analysis was performed (Table 3), with PCOS diagnosis as the dependent variable and tertiles of the total sleep score as independent variables. In Model 1 (unadjusted), compared with the low sleep problem group, the odds ratios (ORs) for PCOS in the medium and high sleep problem groups were 1.787 (95% CI: 1.053–3.031, *p* = 0.031) and 2.490 (95% CI: 1.252–4.951, *p* = 0.009), respectively, indicating a positive association between sleep problem severity and PCOS risk. After adjustment for age, occupation, and educational attainment, the associations remained statistically significant, with ORs of 1.935 (95% CI: 1.106–3.383, *p* = 0.021) and 2.600 (95% CI: 1.263–5.354, *p* = 0.009), consistent with the unadjusted model. Model 2 was further adjusted for BMI, smoking status, and alcohol consumption. Although the magnitude of the association was attenuated, statistical significance was retained. In the fully adjusted model, the ORs for PCOS in the moderate and severe sleep disturbance groups were 1.845 (95% CI: 1.025–3.320, *p* = 0.041) and 2.286 (95% CI: 1.054–4.959, *p* = 0.036), respectively. Overall, greater sleep problem severity was associated with a higher likelihood of PCOS, independent of BMI and other sociodemographic and behavioural factors.

**Table 3:** Multivariate logistic regression analysis of sleep problem severity and the likelihood of polycystic ovary syndrome.

Model	Sleep quality								
	Low			Moderate			High		
	OR	95% CI	p value	OR	95% CI	p value	OR	95% CI	p value
Crude	Ref.			1.787	1.053–3.031	0.031*	2.490	1.252–4.951	0.009**
Model 1				1.935	1.106–3.383	0.021*	2.600	1.263–5.354	0.009**
Model 2				1.845	1.025–3.320	0.041*	2.286	1.054–4.959	0.036*

Note. Binary logistic regression was employed for the analysis. Three models were constructed: the Crude model (unadjusted), Model 1 (adjusted for age, occupation, and educational level), and Model 2 (further adjusted for BMI, smoking history, and drinking history). Participants were categorized based on their total sleep problem scores as follows: “Low” (scores 1–4), “Moderate” (scores 5–9), and “High” (scores ≥ 10).* *p* < 0.05, ** *p* < 0.01.

## Discussion

This study, based on systematic questionnaire surveys of women of reproductive age, demonstrated that after adjustment for key confounding factors, including BMI, sleep problem severity was independently and positively associated with PCOS, and greater sleep problem severity was associated with higher odds of PCOS. The multidimensional sleep assessment questionnaire effectively captured the sleep characteristics of women with PCOS. The strongest associations with PCOS were observed for three specific sleep dimensions: difficulty falling asleep, non-restorative sleep manifested as morning fatigue, and poor subjective sleep quality. These findings provide novel evidence for understanding the complex relationship between PCOS and sleep, suggesting that prevalent subjective sleep disturbances—beyond obesity-related OSA—represent an important yet under-recognised aspect of PCOS management.

Sleep is a fundamental physiological requirement. With ongoing societal development, increasing psychosocial and occupational pressures have contributed to a widespread decline in sleep quality and a growing prevalence of sleep-related problems. Previous studies have reported that more than 60% of women with PCOS experience sleep disturbances^31^. Moreover, women with PCOS who are obese exhibit reduced sleep efficiency^32^. More importantly, sleep quality may further influence the beneficial effects of diet quality on PCOS^33^. Beyond clinical associations, a Mendelian randomisation study provided evidence at the genetic level supporting a causal relationship, demonstrating that genetic susceptibility to insomnia is associated with an increased risk of PCOS^34^. These findings support the notion that sleep disturbances may represent a potential risk factor for PCOS. In light of this, the present study focuses on “subclinical” sleep disturbances, including difficulty falling asleep (sleep onset latency > 30 min) and non-restorative sleep (persistent fatigue upon waking), which do not meet the diagnostic criteria for insomnia. Unlike clinically diagnosed insomnia, these subthreshold sleep disturbances are more prevalent in the general population and may manifest at an earlier stage.The present study found that, even without constituting an independent sleep disorder diagnosis, these complaints themselves are significantly associated with PCOS status. Although the causal direction remains unclear, they warrant some attention in clinical assessment and management.

IR, a core pathological feature of PCOS that often exists independently of obesity, represents a key mechanistic link between sleep and PCOS^35^. IR is not a singular metabolic abnormality, but rather a systemic pathological condition with distinct manifestations across multiple tissues. Within adipose tissue, IR results in impaired lipolysis and excessive lipid accumulation, and concurrently, the hyperinsulinemic state induced by IR promotes increased local androgen synthesis^36–38^. In skeletal muscle, impaired insulin signalling results in reduced postprandial glucose uptake, providing a mechanistic basis for the increased susceptibility of women with PCOS to type 2 diabetes^39–41^. In the liver, IR suppresses the synthesis of sex hormone-binding globulin (SHBG), resulting in elevated circulating free testosterone levels^42–44^. Within the ovaries, this manifests as “selective insulin resistance”, which directly drives hyperandrogenism and ovulatory dysfunction^18,19,45,46^. Moreover, IR can modulate the pulsatile secretion of gonadotropin-releasing hormone (GnRH) in the hypothalamus through central nervous system pathways, thereby disrupting hypothalamic-pituitary-ovarian (HPO) axis function^47^. On the one hand, insufficient sleep represents an independent risk factor for IR. Previous studies have demonstrated that even mild sleep restriction can result in significant increases in fasting insulin levels and the homeostasis model assessment of insulin resistance (HOMA-IR), independent of changes in fat mass^48,49^. On the other hand, IR, a core feature of PCOS, also represents an important pathological basis underlying sleep abnormalities. IR, together with associated chronic low-grade inflammation, oxidative stress, and sympathetic nervous system activation, can substantially increase the risk and severity of OSA through multiple mechanisms, including promoting visceral and upper airway peripharyngeal fat accumulation, inducing inflammatory impairment of pharyngeal muscle function, and disrupting the stability of respiratory control^50^. The pronounced ‘morning fatigue’ (non-restorative sleep) observed in PCOS patients in this study may also stem from impaired sleep quality caused by IR and its associated metabolic disorders, ultimately preventing adequate energy restoration.

The distinctive hormonal milieu associated with PCOS can directly and indirectly influence sleep patterns. Periods characterized by substantial hormonal fluctuations are associated with an increased likelihood of sleep disturbances^51^. For example, sleep disturbances are more prevalent among perimenopausal women, whereas premenopausal women engaged in long night shifts, experiencing short sleep duration, or frequent nocturnal awakenings tend to exhibit relatively elevated levels of estradiol or testosterone^52^. Persistent hormonal imbalances in women with PCOS may contribute to the chronicity of their sleep disturbances. Compared with the general population, women with PCOS frequently exhibit abnormal sex hormone profiles, primarily characterised by elevated androgens, increased luteinising hormone (LH) levels and luteinising hormone/follicle-stimulating hormone ratios, alongside potentially reduced progesterone levels during the luteal phase^53,54^. Although hyperandrogenism is a well-recognised risk factor for OSA^51,55^, the direct relationship between elevated androgen levels and non-apnoeic sleep disturbances remains poorly understood^56^. Among neuroendocrine mechanisms affecting subjective sleep perception, progesterone appears to play a particularly central role. Endogenous progesterone exerts notable anxiolytic and sedative effects, whereas its metabolite, allopregnanolone, functions as a potent positive allosteric modulator of gamma-aminobutyric acid type A receptors in the central nervous system, with comparable efficacy, and both compounds effectively facilitate sleep and contribute to autonomic nervous system stability^6,57,58^. In patients with PCOS, cyclical progesterone deficiency may attenuate inhibitory GABAergic tone, thereby reducing the brain’s regulatory “braking” capacity and increasing susceptibility to hyperarousal states. This mechanism may represent an intrinsic factor contributing to the high prevalence and severity of sleep disturbances in this population. Under physiological conditions, progesterone effectively suppresses the pulsatile frequency of hypothalamic GnRH, thereby regulating LH secretion. However, in PCOS, the hyperandrogenic milieu reduces GnRH neuronal sensitivity to progesterone-mediated negative feedback, resulting in accelerated LH pulsatility^59^. This pattern of high-frequency LH secretion is also closely linked to sleep disturbances^60^. Moreover, the effects of sleep disturbances on PCOS may be mediated through disruption of the circadian rhythm system. Circadian dysregulation and dysfunction of its core regulatory factor, melatonin, constitute a pivotal mechanistic link between sleep disturbances and the pathophysiology of PCOS. Melatonin not only regulates the sleep–wake cycle but also exhibits antioxidant, anti-inflammatory, and immunomodulatory properties^61^. Evidence suggests that melatonin alleviates IR in ovarian granulosa cells (GCs) and promotes the conversion of androgens to oestrogens ^62,63^. In GCs from women with PCOS, binding motifs for circadian-related transcription factors were largely absent, and their expression patterns were partially disrupted^81^. These findings indicate that sleep disturbances may directly impair follicular development and ovarian function by disrupting melatonin signalling and the local ovarian circadian clock, thereby contributing substantially to the pathogenesis and progression of PCOS.

Compared with women without PCOS, most women with PCOS display markedly elevated serum levels of multiple inflammatory markers, placing individuals with long-standing PCOS in a state of chronic systemic inflammation^64–66^. This chronic inflammatory state is primarily linked to visceral adiposity. Hypoxic adipocytes undergo necrosis, recruit inflammatory cells, and release various pro-inflammatory mediators, thereby amplifying systemic inflammatory responses^64,65,67^. At the immune cell level, women with PCOS also display an imbalance in macrophage polarization^68,69^, elevated neutrophil counts and an increased neutrophil-to-lymphocyte ratio^70^, and T lymphocyte subset dysregulation, further driving and sustaining the inflammatory process^71–73^. A large-scale population study using data from the US National Health and Nutrition Examination Survey (NHANES) reported that inflammatory biomarkers—including white blood cell count, neutrophil count, neutrophil-to-lymphocyte ratio, and systemic inflammatory indices—mediate the relationship between sedentary behaviour and sleep disturbances^74^. PCOS may exacerbate disturbances in neuroendocrine balance and sleep-regulatory centres via analogous inflammatory pathways, resulting in impaired sleep quality and shortened sleep duration^64^. Additionally, PCOS-related inflammation may contribute to subjective sleep disturbances, including difficulty initiating sleep and non-restorative sleep manifested as morning fatigue^64,74^. Notably, sleep disturbances may further exacerbate inflammation and metabolic dysregulation through activation of the hypothalamic-pituitary-adrenal (HPA) axis. Animal studies have demonstrated that sleep deprivation, particularly eye movement sleep (REM) sleep deprivation, induces sustained HPA axis activation, with response patterns closely resembling those observed under psychological stress^75^. Persistent HPA axis hyperactivity and dysregulated glucocorticoid secretion, induced by sleep disturbances, may further aggravate IR, chronic inflammation, and hypothalamic-pituitary-gonadal axis dysfunction, thereby promoting the development and persistence of PCOS^75–78^.

The processes involving IR, imbalance of sex hormone receptors and their co-regulators, and chronic low-grade inflammation play important roles in the endometrial pathophysiology of PCOS. These processes interact and synergize, ultimately leading to endometrial dysfunctions in women with PCOS, including progesterone resistance, impaired glucose transport, and abnormal angiogenesis^10^. Concurrently, the aforementioned pathophysiological processes and related mechanisms may alter the ovarian microenvironment through mechanisms such as oxidative stress and lipotoxicity, thereby compromising oocyte development and maturation^79^. This suggests that reproductive dysfunction in PCOS patients may involve a combined impairment at both the endometrial and oocyte levels. In summary, we speculate that PCOS‑related sleep disturbances may participate in this complex pathological process, thus constituting a potential factor that adversely affects endometrial function and oocyte quality.

It is also noteworthy that emotional state constitutes an important mediator of the relationship between sleep disturbances and PCOS. In the present study, non-restorative sleep and poor subjective sleep quality were more prevalent among women with PCOS. Additionally, previous studies have reported strong correlations between insomnia and fatigue^80^, and these sleep disturbances have been associated with elevated levels of anxiety and depressive symptoms. Based on the above findings, we speculate that in PCOS patients, emotional state may simultaneously influence both daytime functioning and subjective sleep experience. Hachui et al.^56^ investigated polysomnograms (PSG) and found that patients with PCOS exhibited significantly reduced periods of REM, suggesting potential impairment in their sleep architecture. REM sleep is crucial for emotional regulation and cognitive function^51^. Among PCOS patients, psychological distress (anxiety/depression) may form a bidirectional relationship with sleep disturbances (particularly fatigue) while also acting as an independent risk factor for PCOS: disrupted sleep architecture exacerbates daytime fatigue and emotional burden, whereas emotional stress can worsen sleep onset difficulties and sleep fragmentation, thereby further compromising sleep quality.

Finally, with respect to the impact of sleep patterns, several studies have reported an association between night shift work and the development of PCOS^81^. It is well established that disrupted circadian rhythms negatively impact health outcomes. However, in the present study, no statistically significant association was observed between bedtime or sleep duration and PCOS. This may be due to the lack of distinction between night-shift and rotating-shift schedules, as well as the limited sample source and size. Therefore, these findings should be interpreted with caution. Nonetheless, these findings have important clinical implications: routine evaluation and long-term management of PCOS should systematically assess subjective sleep complaints, including difficulty initiating sleep and non-restorative sleep. Targeted management of these sleep disturbances may represent a key strategy for interrupting the ‘poor sleep–metabolic–endocrine deterioration’ cycle, enhancing patients’ quality of life, and potentially improving reproductive and metabolic outcomes.

## Limitations and future directions

This study has several objective limitations. First, the simplified questionnaire derived from core items of the PSQI was used for assessment. This instrument effectively captures core dimensions of subjective sleep perception and provides a reasonably accurate reflection of the overall sleep status of PCOS patients. However, it has limitations in evaluating macroscopic sleep architecture (e.g., stage proportions, arousal frequency) and cannot replace objective monitoring techniques such as polysomnography. Future studies should combine subjective and objective methods to provide a more comprehensive sleep profile.

Second, this study did not assess key variables that may influence sleep quality, including mental health variables (e.g., depressive and anxiety symptoms), biochemical markers (IR-related markers, androgen levels, inflammatory markers), as well as physical activity levels and certain sociodemographic information (marital status, reproductive history, living arrangements). The absence of these variables limits interpretation of whether sleep disturbances are independently associated with PCOS itself or are attributable to the underlying metabolic and endocrine abnormalities commonly seen in PCOS, such as IR, hyperandrogenism, and chronic low-grade inflammation. Future studies should address these variables.

Third, as a case-control study, this research can only reveal associations between PCOS and sleep problems without inferring causality, and the sample size was insufficient to support subgroup analyses according to PCOS phenotypes. In addition, the sample was drawn from a single-center outpatient setting, which may introduce selection bias and limit the generalizability of the findings. However, all cases included in this study underwent rigorous clinical diagnosis, ensuring data authenticity and reliability in reflecting the actual sleep status of women with PCOS. Future studies should employ longitudinal designs or experimental interventions to clarify whether sleep disturbances are a cause, a consequence, or bidirectionally related to PCOS.

Nevertheless, as an exploratory study, this research assessed the the association between PCOS and multidimensional subjective sleep parameters while controlling for BMI. The findings provide preliminary observational evidence in this emerging area, laying the groundwork for future, more in-depth studies to elucidate underlying mechanisms and potential interventions.

## Supplementary Information

Below is the link to the electronic supplementary material.

Supplementary Material 1

## Data Availability

All data supporting the findings of this study are available within the paper and its Supplementary Information.

## Abbreviations

PCOS: Polycystic ovary syndrome
BMI: Body mass index
IR: Insulin resistance
aOR: Adjusted odds ratio
OSA: Obstructive sleep apnoea
PSQI: Pittsburgh Sleep Quality Index
SHBG: Sex hormone-binding globulin
GnRH: Gonadotropin-releasing hormone
HPO: Hypothalamic-pituitary-ovarian
HOMA-IR: Homeostasis model assessment of insulin resistance
LH: Luteinising hormone
GCs: Ovarian granulosa cells
HPA: Hypothalamic-pituitary-adrenal
PSG: Polysomnograms
REM: Rapid eye movement sleep

## References

[1] Teede, H. J. et al. Recommendations from the 2023 international evidence-based guideline for the assessment and management of polycystic ovary syndrome. *Eur. J. Endocrinol.* **189**(2), G43-g64. 10.1093/ejendo/lvad096 (2023). 37580861 10.1093/ejendo/lvad096

[2] Azziz, R. et al. Polycystic ovary syndrome. *Nat. Rev. Dis. Primers.* **2**, 16057. 10.1038/nrdp.2016.57 (2016). 27510637 10.1038/nrdp.2016.57

[3] Escobar-Morreale, H. F. Polycystic ovary syndrome: Definition, aetiology, diagnosis and treatment. *Nat. Rev. Endocrinol.* **14**(5), 270–84. 10.1038/nrendo.2018.24 (2018). 29569621 10.1038/nrendo.2018.24

[4] Stener-Victorin, E. et al. Polycystic ovary syndrome. *Nat. Rev. Dis. Primers.* **10**(1), 27. 10.1038/s41572-024-00511-3 (2024). 38637590 10.1038/s41572-024-00511-3

[5] Aziz, M. et al. Polycystic ovary syndrome: Cardiovascular risk factors according to specific phenotypes. *Acta Obstet. Gynecol. Scand.* **94**(10), 1082–9. 10.1111/aogs.12706 (2015). 26123797 10.1111/aogs.12706

[6] Diamanti-Kandarakis, E. & Dunaif, A. Insulin resistance and the polycystic ovary syndrome revisited: An update on mechanisms and implications. *Endocr. Rev.* **33**(6), 981–1030. 10.1210/er.2011-1034 (2012). 23065822 10.1210/er.2011-1034 PMC5393155

[7] Livadas, S., Anagnostis, P., Bosdou, J. K., Bantouna, D. & Paparodis, R. Polycystic ovary syndrome and type 2 diabetes mellitus: A state-of-the-art review. *World J. Diabetes.* **13**(1), 5–26. 10.4239/wjd.v13.i1.5 (2022). 35070056 10.4239/wjd.v13.i1.5 PMC8771268

[8] Gao, L. et al. Polycystic ovary syndrome fuels cardiovascular inflammation and aggravates ischemic cardiac injury. *Circulation* **148**(24), 1958–73. 10.1161/circulationaha.123.065827 (2023). 37937441 10.1161/CIRCULATIONAHA.123.065827 PMC10713005

[9] Palomba, S. et al. Pregnancy complications in women with polycystic ovary syndrome. *Hum. Reprod. Update.* **21**(5), 575–92. 10.1093/humupd/dmv029 (2015). 26117684 10.1093/humupd/dmv029

[10] Palomba, S., Piltonen, T. T. & Giudice, L. C. Endometrial function in women with polycystic ovary syndrome: A comprehensive review. *Hum. Reprod. Update.* **27**(3), 584–618. 10.1093/humupd/dmaa051 (2021). 33302299 10.1093/humupd/dmaa051

[11] Matsuyama, S., Whiteside, S. & Li, S. Y. Implantation and decidualization in PCOS: Unraveling the complexities of pregnancy. *Int. J. Mol. Sci.*10.3390/ijms25021203 (2024). 38256276 10.3390/ijms25021203 PMC10816633

[12] Bahri Khomami, M. et al. Systematic review and meta-analysis of pregnancy outcomes in women with polycystic ovary syndrome. *Nat. Commun.* **15**(1), 5591. 10.1038/s41467-024-49749-1 (2024). 38965226 10.1038/s41467-024-49749-1 PMC11224312

[13] Cooney, L. G., Lee, I., Sammel, M. D. & Dokras, A. High prevalence of moderate and severe depressive and anxiety symptoms in polycystic ovary syndrome: a systematic review and meta-analysis. *Hum. Reprod.* **32**(5), 1075–91. 10.1093/humrep/dex044 (2017). 28333286 10.1093/humrep/dex044

[14] Karjula, S. et al. Psychological distress is more prevalent in fertile age and premenopausal women with PCOS symptoms: 15-year follow-up. *J. Clin. Endocrinol. Metab.* **102**(6), 1861–9. 10.1210/jc.2016-3863 (2017). 28323926 10.1210/jc.2016-3863 PMC5470769

[15] Merkin, S. S., Phy, J. L., Sites, C. K. & Yang, D. Environmental determinants of polycystic ovary syndrome. *Fertil. Steril.* **106**(1), 16–24. 10.1016/j.fertnstert.2016.05.011 (2016). 27240194 10.1016/j.fertnstert.2016.05.011

[16] Batra, M., Bhatnager, R., Kumar, A., Suneja, P. & Dang, A. S. Interplay between PCOS and microbiome: the road less travelled. *Am. J. Reprod. Immunol.* **88**(2), e13580. 10.1111/aji.13580 (2022). 35598286 10.1111/aji.13580

[17] Singh, S. et al. Polycystic ovary syndrome: Etiology, current management, and future therapeutics. *J. Clin. Med.*10.3390/jcm12041454 (2023). 36835989 10.3390/jcm12041454 PMC9964744

[18] Stańczak, N. A., Grywalska, E. & Dudzińska, E. The latest reports and treatment methods on polycystic ovary syndrome. *Ann. Med.* **56**(1), 2357737. 10.1080/07853890.2024.2357737 (2024). 38965663 10.1080/07853890.2024.2357737 PMC11229724

[19] Wang, J., Wu, D., Guo, H. & Li, M. Hyperandrogenemia and insulin resistance: The chief culprit of polycystic ovary syndrome. *Life Sci.* **236**, 116940. 10.1016/j.lfs.2019.116940 (2019). 31604107 10.1016/j.lfs.2019.116940

[20] Palomba, S. Is fertility reduced in ovulatory women with polycystic ovary syndrome? An opinion paper. *Hum. Reprod.* **36**(9), 2421–8. 10.1093/humrep/deab181 (2021). 34333641 10.1093/humrep/deab181

[21] Huber-Buchholz, M. M., Carey, D. G. & Norman, R. J. Restoration of reproductive potential by lifestyle modification in obese polycystic ovary syndrome: Role of insulin sensitivity and luteinizing hormone. *J. Clin. Endocrinol. Metab.* **84**(4), 1470–4. 10.1210/jcem.84.4.5596 (1999). 10199797 10.1210/jcem.84.4.5596

[22] Crosignani, P. G. et al. Overweight and obese anovulatory patients with polycystic ovaries: Parallel improvements in anthropometric indices, ovarian physiology and fertility rate induced by diet. *Hum. Reprod.* **18**(9), 1928–32. 10.1093/humrep/deg367 (2003). 12923151 10.1093/humrep/deg367

[23] Han, Y., Liu, Z. & Chen, T. Role of vaginal microbiota dysbiosis in gynecological diseases and the potential interventions. *Front. Microbiol.* **12**, 643422. 10.3389/fmicb.2021.643422 (2021). 34220737 10.3389/fmicb.2021.643422 PMC8249587

[24] Dou, Z., Li, Q., Zhang, J. & Zhang, X. Exploring the mechanism of *Schisandra rubriflora* in the treatment of polycystic ovary syndrome based on network pharmacology and molecular docking. *J. Ovarian Res.* **18**(1), 16. 10.1186/s13048-025-01600-x (2025). 39875917 10.1186/s13048-025-01600-x PMC11773789

[25] Layegh, P., Mousavi, Z., Farrokh Tehrani, D., Parizadeh, S. M. & Khajedaluee, M. Insulin resistance and endocrine-metabolic abnormalities in polycystic ovarian syndrome: Comparison between obese and non-obese PCOS patients. *Int. J. Reprod. Biomed.* **14**(4), 263–270. (2016). 27351028 PMC4918775

[26] Abdul Jafar, N. K. et al. Obstructive sleep apnea syndrome in polycystic ovary syndrome: a systematic review and meta-analysis. *Front Endocrinol (Lausanne)* **16**, 1532519. 10.3389/fendo.2025.1532519 (2025). 40255502 10.3389/fendo.2025.1532519 PMC12006010

[27] Decrinis, C. et al. Sleep disorders and psychological comorbidities in women with polycystic ovary syndrome—a cross-sectional study. *Arch. Gynecol. Obstet.* **312**(2), 573–82. 10.1007/s00404-025-08049-9 (2025). 40358729 10.1007/s00404-025-08049-9 PMC12334535

[28] Hu, P., Vinturache, A., Chen, Y., Ding, G. & Zhang, Y. Joint Association of Sleep Onset Time and Sleep Duration With Cardiometabolic Health Outcome. *J. Am. Heart Assoc.* **13** (12), e034165. 10.1161/jaha.123.034165 (2024). 38874059 10.1161/JAHA.123.034165 PMC11255762

[29] Fu, R. et al. Association between the intake of potentially risky beverages and the occurrence of endometrial polyps: a case-control study. *Front. Nutr.* **12**, 1538405. 10.3389/fnut.2025.1538405 (2025). 39968393 10.3389/fnut.2025.1538405 PMC11832405

[30] Cui, Y. et al. Systematic review and meta-analysis of animal experiments on Bushen formulas for the treatment of polycystic ovary syndrome. *Sci. Rep.* **15** (1), 7648. 10.1038/s41598-025-89725-3 (2025). 40038344 10.1038/s41598-025-89725-3 PMC11880369

[31] Fernandez, R. C. et al. Sleep disturbances in women with polycystic ovary syndrome: prevalence, pathophysiology, impact and management strategies. *Nat. Sci. Sleep.* **10**, 45–64. 10.2147/nss.S127475 (2018). 29440941 10.2147/NSS.S127475 PMC5799701

[32] Oberg, E., Blomberg, L., Åkerstedt, T. & Hirschberg, A. L. Different sleep pattern in over-weight/obese women with polycystic ovary syndrome. *Front. Endocrinol. (Lausanne)*. **14**, 1068045. 10.3389/fendo.2023.1068045 (2023). 36843616 10.3389/fendo.2023.1068045 PMC9950253

[33] Bennett, C. J. et al. Sleep disturbances may influence lifestyle behaviours in women with self-reported polycystic ovary syndrome. *Br. J. Nutr*. 10.1017/s0007114521002361(2021). 34176543 10.1017/S0007114521002361

[34] Fang, L. et al. Insomnia and female reproductive diseases: a cross-sectional and mendelian randomization study. *Int. J. Womens Health*. **17**, 439–447. (2025). 10.2147/ijwh.S498231 39990928 10.2147/IJWH.S498231 PMC11846518

[35] Li, X. et al. The degree of menstrual disturbance is associated with the severity of insulin resistance in PCOS. *Front. Endocrinol. (Lausanne)*. **13**, 873726 10.3389/fendo.2022.873726. (2022). 35769085 10.3389/fendo.2022.873726 PMC9235353

[36] O’Reilly, M. W. et al. AKR1C3-mediated adipose androgen generation drives lipotoxicity in women with polycystic ovary syndrome. *J. Clin. Endocrinol. Metab.* **102** (9), 3327–3339. 10.1210/jc.2017-00947 (2017). 28645211 10.1210/jc.2017-00947 PMC5587066

[37] Corbould, A. Chronic testosterone treatment induces selective insulin resistance in subcutaneous adipocytes of women. *J. Endocrinol.* **192**(3), 585–94. 10.1677/joe.1.07070 (2007). 17332526 10.1677/joe.1.07070

[38] Paulukinas, R. D., Mesaros, C. A. & Penning, T. M. Conversion of classical and 11-oxygenated androgens by insulin-induced AKR1C3 in a model of human PCOS adipocytes. *Endocrinology*10.1210/endocr/bqac068 (2022). 35560164 10.1210/endocr/bqac068 PMC9162389

[39] Corbould, A. et al. Insulin resistance in the skeletal muscle of women with PCOS involves intrinsic and acquired defects in insulin signaling. *Am. J. Physiol. Endocrinol. Metab.* **288**(5), E1047-54. 10.1152/ajpendo.00361.2004 (2005). 15613682 10.1152/ajpendo.00361.2004

[40] Corbould, A., Zhao, H., Mirzoeva, S., Aird, F. & Dunaif, A. Enhanced mitogenic signaling in skeletal muscle of women with polycystic ovary syndrome. *Diabetes* **55**(3), 751–9. 10.2337/diabetes.55.03.06.db05-0453 (2006). 16505239 10.2337/diabetes.55.03.06.db05-0453

[41] Højlund, K. et al. Impaired insulin-stimulated phosphorylation of Akt and AS160 in skeletal muscle of women with polycystic ovary syndrome is reversed by pioglitazone treatment. *Diabetes* **57**(2), 357–66. 10.2337/db07-0706 (2008). 17977950 10.2337/db07-0706

[42] Deswal, R., Yadav, A. & Dang, A. S. Sex hormone binding globulin—an important biomarker for predicting PCOS risk: a systematic review and meta-analysis. *Syst. Biol. Reprod. Med.* **64**(1), 12–24. 10.1080/19396368.2017.1410591 (2018). 29227165 10.1080/19396368.2017.1410591

[43] Cui, P. et al. Long-term androgen excess induces insulin resistance and non-alcoholic fatty liver disease in PCOS-like rats. *J. Steroid. Biochem. Mol. Biol.* **208**, 105829. 10.1016/j.jsbmb.2021.105829 (2021). 33513383 10.1016/j.jsbmb.2021.105829

[44] Spremović Rađenović, S. et al. Prevalence, risk factors, and pathophysiology of nonalcoholic fatty liver disease (NAFLD) in women with polycystic ovary syndrome (PCOS). *Biomedicines*10.3390/biomedicines10010131 (2022). 35052811 10.3390/biomedicines10010131 PMC8773533

[45] Sanchez-Garrido, M. A. & Tena-Sempere, M. Metabolic dysfunction in polycystic ovary syndrome: Pathogenic role of androgen excess and potential therapeutic strategies. *Mol. Metab.* **35**, 100937. 10.1016/j.molmet.2020.01.001 (2020). 32244180 10.1016/j.molmet.2020.01.001 PMC7115104

[46] Zhang, C., Hu, J., Wang, W., Sun, Y. & Sun, K. HMGB1-induced aberrant autophagy contributes to insulin resistance in granulosa cells in PCOS. *FASEB J.* **34**(7), 9563–74. 10.1096/fj.202000605RR (2020). 32469087 10.1096/fj.202000605RR

[47] Li, S. et al. Research progress on the effect of epilepsy and antiseizure medications on PCOS through HPO axis. *Front Endocrinol (Lausanne)* **12**, 787854. 10.3389/fendo.2021.787854 (2021). 34992582 10.3389/fendo.2021.787854 PMC8726549

[48] Zuraikat, F. M. et al. Chronic insufficient sleep in women impairs insulin sensitivity independent of adiposity changes: Results of a randomized trial. *Diabetes Care* **47**(1), 117–25. 10.2337/dc23-1156 (2024). 37955852 10.2337/dc23-1156 PMC10733650

[49] Widjaja, N. A., Kurube, C. F. & Ardianah, E. Sleep duration and insulin resistance in obese adolescents with metabolic syndrome: Is there a correlation?. *Acta Biomed.* **94**(4), e2023079. 10.23750/abm.v94i4.14142 (2023). 37539611 10.23750/abm.v94i4.14142 PMC10440761

[50] Yin, H., Huang, W. & Yang, B. Association between METS-IR index and obstructive sleep apnea: evidence from NHANES. *Sci. Rep.* **15**(1), 6654. 10.1038/s41598-024-84040-9 (2025). 39994225 10.1038/s41598-024-84040-9 PMC11850641

[51] Haufe, A. & Leeners, B. Sleep disturbances across a woman’s lifespan: What is the role of reproductive hormones?. *J. Endocr. Soc.* **7**(5), bvad036. 10.1210/jendso/bvad036 (2023). 37091307 10.1210/jendso/bvad036 PMC10117379

[52] Nagata, C. et al. Sleep-related factors and circulating levels of sex hormones in premenopausal Japanese women. *Endocr. J.* **70**(3), 267–73. 10.1507/endocrj.EJ22-0337 (2023). 36384969 10.1507/endocrj.EJ22-0337

[53] Yang, Y., Liu, B., Wu, G. & Yang, J. Exploration of the value of progesterone and progesterone/estradiol ratio on the hCG trigger day in predicting pregnancy outcomes of PCOS patients undergoing IVF/ICSI: a retrospective cohort study. *Reprod. Biol. Endocrinol.* **19**(1), 184. 10.1186/s12958-021-00862-6 (2021). 34893087 10.1186/s12958-021-00862-6 PMC8665570

[54] Zhang, H. et al. Relationship between body composition, insulin resistance, and hormonal profiles in women with polycystic ovary syndrome. *Front. Endocrinol. (Lausanne)*. **13**, 1085656. 10.3389/fendo.2022.1085656 (2022). 36699018 10.3389/fendo.2022.1085656 PMC9869160

[55] Jafar, N. K. A., Fan, M., Moran, L. J., Mansfield, D. R. & Bennett, C. J. Sex hormones, sex hormone-binding globulin and sleep problems in females with polycystic ovary syndrome: a systematic review and meta-analysis. *Clin Endocrinol (Oxf)* **102**(6), 708–20. 10.1111/cen.15219 (2025). 39996383 10.1111/cen.15219 PMC12046544

[56] Hachul, H. et al. Sleep disorders in polycystic ovary syndrome: influence of obesity and hyperandrogenism. *Rev. Assoc. Med. Bras.* **65**(3), 375–83. 10.1590/1806-9282.65.3.375 (2019). 30994836 10.1590/1806-9282.65.3.375

[57] Hantsoo, L. & Epperson, C. N. Allopregnanolone in premenstrual dysphoric disorder (PMDD): Evidence for dysregulated sensitivity to GABA-A receptor modulating neuroactive steroids across the menstrual cycle. *Neurobiol. Stress.* **12**, 100213. 10.1016/j.ynstr.2020.100213 (2020). 32435664 10.1016/j.ynstr.2020.100213 PMC7231988

[58] Brown, R. D., Bondy, E., Prim, J., Dichter, G. & Schiller, C. E. The behavioral and physiological correlates of affective mood switching in premenstrual dysphoric disorder. *Front. Psychiatry.* **15**, 1448914. 10.3389/fpsyt.2024.1448914 (2024). 39559281 10.3389/fpsyt.2024.1448914 PMC11570288

[59] Pielecka, J., Quaynor, S. D. & Moenter, S. M. Androgens increase gonadotropin-releasing hormone neuron firing activity in females and interfere with progesterone negative feedback. *Endocrinology* **147**(3), 1474–9. 10.1210/en.2005-1029 (2006). 16339200 10.1210/en.2005-1029

[60] Mohammadi, H., Rezaei, M., Faghihi, F. & Khazaie, H. Hypothalamic-pituitary-gonadal activity in paradoxical and psychophysiological insomnia. *J. Med. Signals Sens.* **9**(1), 59–67. 10.4103/jmss.JMSS_31_18 (2019). 30967991 10.4103/jmss.JMSS_31_18 PMC6419559

[61] Patel, A., Dewani, D., Jaiswal, A., Yadav, P. & Reddy, L. S. Exploring melatonin’s multifaceted role in polycystic ovary syndrome management: a comprehensive review. *Cureus* **15**(11), e48929. 10.7759/cureus.48929 (2023). 38106751 10.7759/cureus.48929 PMC10725523

[62] Guo, R. et al. Melatonin alleviates insulin resistance through the PI3K/AKT signaling pathway in ovary granulosa cells of polycystic ovary syndrome. *Reprod. Biol.* **22**(1), 100594. 10.1016/j.repbio.2021.100594 (2022). 34953312 10.1016/j.repbio.2021.100594

[63] Yu, K. et al. Melatonin reduces androgen production and upregulates heme oxygenase-1 expression in granulosa cells from PCOS patients with hypoestrogenia and hyperandrogenia. *Oxid. Med. Cell. Longev.* **2019**, 8218650. 10.1155/2019/8218650 (2019). 31772710 10.1155/2019/8218650 PMC6854986

[64] Dăneasă, A. et al. Letrozole vs estradiol valerate induced PCOS in rats: glycemic, oxidative and inflammatory status assessment. *Reproduction* **151** (4), 401–409. 10.1530/rep-15-0352 (2016). 26792865 10.1530/REP-15-0352

[65] Rudnicka, E. et al. Chronic low grade inflammation in pathogenesis of PCOS. *Int. J. Mol. Sci.*10.3390/ijms22073789. (2021). 33917519 10.3390/ijms22073789 PMC8038770

[66] Xiang, Y. et al. Hyperandrogenism drives ovarian inflammation and pyroptosis: a possible pathogenesis of PCOS follicular dysplasia. *Int. Immunopharmacol.* **125** (Pt A), 111141.10.1016/j.intimp.2023.111141 (2023). 37918087 10.1016/j.intimp.2023.111141

[67] Foroozanfard, F., Soleimani, A., Arbab, E., Samimi, M. & Tamadon, M. R. Relationship between IL-17 serum level and ambulatory blood pressure in women with polycystic ovary syndrome. *J. Nephropathol*. **6** (1), 15–24. 10.15171/jnp.2017.04 (2017). 28042549 10.15171/jnp.2017.04 PMC5106878

[68] Qi, X. et al. Hyperhomocysteinemia promotes insulin resistance and adipose tissue inflammation in PCOS mice through modulating M2 macrophage polarization via estrogen suppression. *Endocrinology* **158** (5), 1181–1193. 10.1210/en.2017-00039 (2017). 28323956 10.1210/en.2017-00039

[69] Xie Q, Xiong X, Xiao N, He K, Chen M, Peng J, et al. Mesenchymal stem cells alleviate DHEA-induced polycystic ovary syndrome (PCOS) by inhibiting inflammation in mice. *Stem Cells Int.* **2019**, 9782373. 10.1155/2019/9782373 (2019). 10.1155/2019/9782373 PMC6757294 31611920

[70] Herlihy, A. C. et al. Polycystic ovary syndrome and the peripheral blood white cell count. *J. Obstet. Gynaecol.* **31** (3), 242–244. 10.3109/01443615.2011.553693 (2011). 21417649 10.3109/01443615.2011.553693

[71] Matteo, M. et al. Reduced percentage of natural killer cells associated with impaired cytokine network in the secretory endometrium of infertile women with polycystic ovary syndrome. *Fertil Steril.* ;**94**(6):2222–222. 10.1016/j.fertnstert.2010.01.049 7.e1–3. (2010). 20236632 10.1016/j.fertnstert.2010.01.049

[72] Krishna, M. B. et al. Reduced Tregs in peripheral blood of PCOS patients—a consequence of aberrant Il2 signaling. *J. Clin. Endocrinol. Metab.* **100** (1), 282–292. 10.1210/jc.2014-2401 (2015). 25303485 10.1210/jc.2014-2401

[73] Ma, M., Wang, M., Xu, F. & Hao, S. The imbalance in Th17 and Treg cells in polycystic ovarian syndrome patients with autoimmune thyroiditis. *Immunol. Invest.* **51**(5), 1170–81. 10.1080/08820139.2021.1915329 (2022). 33902382 10.1080/08820139.2021.1915329

[74] You, Y. et al. The association between sedentary behavior, exercise, and sleep disturbance: a mediation analysis of inflammatory biomarkers. *Front. Immunol.* **13**, 1080782. 10.3389/fimmu.2022.1080782 (2022). 36713451 10.3389/fimmu.2022.1080782 PMC9880546

[75] Moraes, D. A., Machado, R. B., Koban, M., Hoffman, G. E. & Suchecki, D. The pituitary-adrenal response to paradoxical sleep deprivation is similar to a psychological stressor, whereas the hypothalamic response is unique. *Front Endocrinol (Lausanne)* **13**, 885909. 10.3389/fendo.2022.885909 (2022). 35880052 10.3389/fendo.2022.885909 PMC9308007

[76] Sapolsky, R. M., Romero, L. M. & Munck, A. U. How do glucocorticoids influence stress responses? Integrating permissive, suppressive, stimulatory, and preparative actions. *Endocr. Rev.* **21**(1), 55–89. 10.1210/edrv.21.1.0389 (2000). 10696570 10.1210/edrv.21.1.0389

[77] Yang, Y. et al. Causal associations between sleep traits and four cardiac diseases: a mendelian randomization study. *ESC Heart Fail.* **9**(5), 3160–6. 10.1002/ehf2.14016 (2022). 35765714 10.1002/ehf2.14016 PMC9715768

[78] Mostafa, S. A. et al. Sleep behaviours and associated habits and the progression of pre-diabetes to type 2 diabetes mellitus in adults: a systematic review and meta-analysis. *Diab. Vasc. Dis. Res.* **19**(3), 14791641221088824. 10.1177/14791641221088824 (2022). 35616501 10.1177/14791641221088824 PMC9152198

[79] Palomba, S., Daolio, J. & La Sala, G. B. Oocyte competence in women with polycystic ovary syndrome. *Trends Endocrinol. Metab.* **28**(3), 186–98. 10.1016/j.tem.2016.11.008 (2017). 27988256 10.1016/j.tem.2016.11.008

[80] Bajpai, A. & Sharma, D. Psychological distress and fatigue in polycystic ovarian syndrome: a pilot study. *J. Hum. Reprod. Sci.* **18**(2), 113–7. 10.4103/jhrs.jhrs_25_25 (2025). 40740626 10.4103/jhrs.jhrs_25_25 PMC12306717

[81] Wang, F. et al. Association between circadian rhythm disruption and polycystic ovary syndrome. *Fertil. Steril.* **115**(3), 771–81. 10.1016/j.fertnstert.2020.08.1425 (2021). 33358334 10.1016/j.fertnstert.2020.08.1425

