# Supplementary material for: A case control study of subjective sleep characteristics and polycystic ovary syndrome
Source: Sci Rep. 2026 Apr 29;16:15053. doi: 10.1038/s41598-026-50477-3 (PMC13172497; doi:10.1038/s41598-026-50477-3)
Supplement: Supplementary file 1 — Supplementary Material 1 [file 41598_2026_50477_MOESM1_ESM.docx]

**Survey Questionnaire**

**1. Your age (years):**
[________________]

**2. What is your ethnicity?***:*

- Han ethnicity
- Other ethnicity

**3. What is your occupation?**

- Professional/Technical
- Service Sector
- Manual Laborer
- Student
- Homemaker/Freelancer
- Manager/Civil Servant
- Unemployed
- Other

**4. Your educational attainment:**

- Junior high school or below
- High school
- University or above

**5. Your place of residence** *(Considering environmental pollution):*

- Urban area (city)
- Town (township)
- Rural area (countryside)

**6. Your height (m):**
[________________]

**7. Your weight (kg):**
[________________]

**8. Have you been clinically diagnosed with polycystic ovary syndrome (PCOS)?**

- Yes
- No

**9. Do you smoke?**

- Yes
- No

**10. Do you drink alcohol?**

- Yes
- No

**Sleep Status Assessment *(Please fill in the information based on your sleep patterns in the past month.)***

**1. What is your average daily bedtime?**

- Before 10:00 PM
- 10:00 PM – 11:00 PM
- 11:00 PM – 12:00 AM
- 12:00 AM – 1:00 AM
- After 1:00 AM

**2. What is your average daily sleep duration?**

- More than 8 hours
- 7 – 8 hours
- 6 – 7 hours
- Less than 6 hours

**3. Do you have difficulty falling asleep (taking more than 30 minutes to fall asleep after going to bed)?**

- Yes
- No

**4. Do you experience nocturnal awakenings?**

- Yes
- No

**5. Do you experience early morning awakenings with inability to resume sleep?**

- Yes
- No

**6. Do you feel fatigued upon waking in the morning (non-restorative sleep)?**

- Yes
- No

**7.  Do you experience vivid dreaming?**

- Yes
- No

**8.  Do you consider your recent sleep quality to be good?**

- Yes
- No
